# Effects of short- and long-term plant functional group removal on alpine meadow community niche

**DOI:** 10.3389/fpls.2024.1474272

**Published:** 2024-11-14

**Authors:** Jingjing Wei, Zhonghua Zhang, Li Ma, Xue Hu, Haze Ade, Hongye Su, Zhengchen Shi, Honglin Li, Huakun Zhou

**Affiliations:** ^1^ Qinghai Provincial Key Laboratory of Restoration Ecology in Cold Regions, Northwest Institute of Plateau Biology, Chinese Academy of Sciences, Xining, China; ^2^ College of Life Sciences, University of Chinese Academy of Sciences, Beijing, China

**Keywords:** biodiversity loss, niche, plant functional group removal, interspecific association, community stability

## Abstract

The rapid loss of global biodiversity affects the creation and maintenance of community biodiversity and ecosystem structure and function. Thus, it is insufficient to focus solely on the effects of biodiversity loss on community biodiversity without also considering other impacts such as community assembly, niches, interspecific relationships, community stability, and biodiversity–ecosystem function. In this study, a 3- and 10-year biodiversity manipulation experiment was conducted in an alpine meadow to examine the effects of the individual plant functional group (PFG) removal on the niches of community dominant species by removal of Gramineae, Cyperaceae, legumes, and other forbs. The results indicated that PFG removal led to variation in community niches. The long-term PFG removal led to a gradual decline in the number of Gramineae and Cyperaceae species in the community. Over time, the niche widths of dominant Gramineae and Cyperaceae species gradually narrowed, and the degree of niche overlapping decreased. The number of positively and negatively associated species tended to decrease and increase, respectively. Reduced species diversity led to significant differences in the niches of the remaining species within the community. Thus, species niche differences, resulting from variation in resource allocation, commonly determined the dynamic construction of species composition within the community.

## 1 Introduction

Species extinction and community simplification threaten ecosystem productivity and services, making it crucial to understand species diversity patterns and processes for effective ecosystem management and conservation (Liang et al., 2015). Plant diversity loss can alter community vegetation characteristics, with responses generally varying based on plant species or functional groups (PFGs) (Chen et al., 2016; Zhang et al., 2017; Fanin et al., 2019). Following disturbances, secondary forbs often replace primary dominant species as new constructive elements (Wang et al., 2009; Li et al., 2013; Zhou et al., 2021). Experiments removing certain PFGs indicated that the target species loss differentially affects aboveground and belowground components in grassland ecosystems, including plant biomass, species richness, soil microorganisms, and soil animals. Studies have shown that the diversity of structural composition within plant communities is vital, as grasslands with lower diversity exhibited reduced soil nutrient retention, making them more vulnerable to changes from species loss (Chen et al., 2016, 2022). The impact of species loss on soil ecosystem functioning varies geographically, although similar contexts can differ significantly (Fanin et al., 2013, 2019). These findings indicated that belowground functions may be more sensitive to environmental changes than plant productivity (Fanin et al., 2019). Species interrelationships and ecosystem function differences are influenced by species selectivity and complementarity (Holt, 2009; Godoy et al., 2020).

A niche represents the minimal needs met by a species and its average impact on environmental conditions. This core theory explains population positions within a community’s ecological space (i.e., resource use) and the dynamics of interspecific coexistence and competition (Loke and Chisholm, 2023). The prevailing hypothesis suggested that niche distinctions foster complementary effects that promote species coexistence when intraspecific competition outstrips interspecific competition (Tilman et al., 1997; Loreau and Hector, 2001; Fox, 2003). Generally, species competition or adaptation drives selection (Chesson, 2000). Community complementarity and niche divergence may arise from interspecific differences in resource use (Barry et al., 2019). Key factors facilitating these differences include selection and complementary effects, influenced by the relative abundance and density of populations, with density effects also shaped by niche differences (Maherali and Klironomos, 2007; Levine and HilleRisLambers, 2009; Godoy et al., 2014). Complementary effects occur when interspecific competition is diminished compared to intraspecific competition, illustrating niche differences (Carroll et al., 2011; Turnbull et al., 2013). When low-productivity species enhance ecosystem functions, negative selection may arise because of reduced interspecific competition, resulting in niche variances (Godoy et al., 2020). Prior studies suggested that greater niche distinctions can foster complementary effects, lessen competition, and increase community homogeneity and stability (Loke and Chisholm, 2023). Community stabilization, which promotes niche differentiation, has been a focal point of coexistence research (Tan et al., 2013). Godoy et al. (2020) found that species exhibit similar sensitivity to competition when balancing intraspecific and interspecific responses. Thus, niche differentiation may further contribute to community equilibrium, with related research indicating that niche divergence enhances resource utilization, leading to increased productivity (Cardinale et al., 2007; Weih et al., 2021).

During short-term species loss, reduced energy in the community may primarily drive changes in plant community structure, particularly leading to decreased productivity (Ward et al., 2009). A decline in community biomass over a short period can result in litter decomposition, impacting soil and species biodiversity (Chen et al., 2016). In contrast, during long-term species loss, niche complementarity likely drives changes in community structure, indicating that species-rich communities more effectively access and utilize limited resources due to their diverse ecological traits. Such ecosystems are considered functionally complete because species complement one another, optimizing resource use (Godoy et al., 2020). It is generally assumed that this complementarity arises from niche differences, which facilitate species coexistence (Buche et al., 2022).

Niche width and niche overlap are key characteristics of species niches (Pielou, 1972). Niche width quantitatively represents the resources available to species, whereas niche overlap refers to competition among different populations for the same resources (Pielou, 1972). Interspecific association denotes the spatial distribution correlations among species, with positive and negative associations indicating their interdependence (Shao and Zhang, 2021). Understanding niche changes is crucial for clarifying the spatiotemporal dynamics of species in communities and competitive exclusion (Lambers et al., 2022; Loke and Chisholm, 2023). Additionally, studying interspecific associations helps to determine the principles governing plant community niches and development, providing a theoretical foundation for species restoration and reconstruction (Huan et al., 2019).

Alpine grasslands are fragile and sensitive to climate change and human activities, making it crucial to understand how species diversity loss affects community stability. We examined the short-term and long-term impacts of specific PFG removal on the niches of remaining species and how species interrelationships change after removal. We separately analyzed data for treatment periods of 3 years (2012–2015) and 10 years (2012–2022) to compare short- and long-term effects of PFG removal. Our objectives were to determine (1) how PFG removal influenced the niches of dominant species and (2) how it affected the trends of community constructive species. Our study aims to deepen understanding of the impact of biodiversity loss on the stability of terrestrial ecosystems.

## 2 Materials and methods

### 2.1 Study area

**Figure 1:**
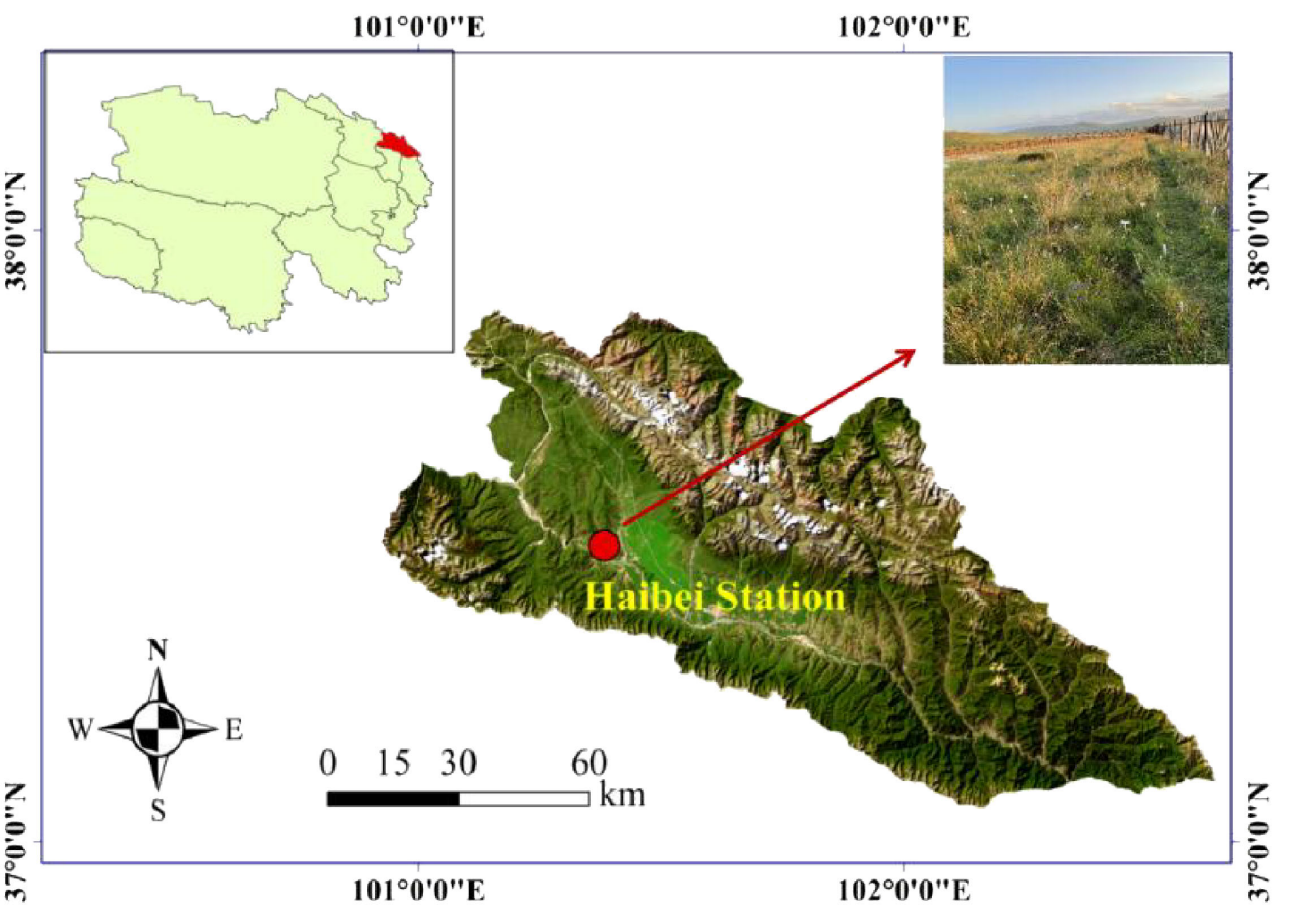
Study area location and site characteristics (satellite layer data source: http://www.locaspace.cn/).

The research site was located at the Haibei Alpine Meadow Ecosystem Research Station (37°29′–37°45′N, 101°12′–101°23′E; 3,200–3,600 m a.s.l.), Menyuan County, Qinghai Province, China (**Figure 1**). The station is characterized by a typical continental plateau climate, with cool, short summers and long, cold winters, a mean temperature of −1.7°C, and a mean annual precipitation of 580 mm (Chen et al., 2022). The local soil is gelic cambisol, which has relatively homogeneous physiochemical properties. The main soil body type is alpine meadow soil, which is a shallow layer of young, weakly alkaline soil formed through simple soil formation processes that is rich in stored nutrients but poor in fast-acting nutrients (Zhang et al., 2017).

### 2.2 PFG removal experiment

The experiment was established on a flat and undisturbed site in 2012. For conservation purposes and to enable regular experimentation, the site was fenced. The targeted species were divided into four PFGs based on ecophysiological characteristics potentially associated with response variables, such as biomass production, resource use patterns, and nitrogen (N) fixation ability (Reich et al., 2001; Gross et al., 2007; McLaren and Turkington, 2010). Cyperaceae are the dominant species in the alpine meadow. Gramineae species exhibit a resource conservation strategy due to their high C:N ratio and leaf dry matter content (Chen et al., 2022). Legumes and other forbs promote species and functional diversity, offering different plant resource utilization pathways that enhance nutrient use and release, leading to higher N and P content in alpine plant communities (Chen et al., 2022). By contrast, species and functional diversity can be fostered by the growth of forbs and legumes, which provide access strategies to improve species resource use and release, leading to higher foliar N and P contents (Chen et al., 2022). In the study area, the species are typical of subalpine meadows, dominated by Cyperaceae, with a diverse forb community. Other PFGs such as Gramineae and legume species are rare, with about 10% Gramineae and 8% of the total coverage. The most abundant plant species are *Carex alatauensis* and *Carex parva* (Cyperaceae), accounting for 40% of the total coverage. All the rest are forbs.

The establishment of the Meadow Removal Experiment (IMGRE) at the Haibei Station was described by Chen et al. (2022). The experiment followed a completely randomized design. Five treatments were established, which include the control (CK), Gramineae removal (RG), Cyperaceae removal (RC), legumes removal (RL), and other forbs removal (RF). Each of the five treatment plots (1 m × 0.75 m) was replicated five times with a total of 25 plots spaced 1 m apart. To minimize physical disturbance to soil, plants were removed by cutting aboveground sections and tiller nodes to 3 cm soil depth, twice per month from May to August (Naeem, 2002; Chen et al., 2016, 2022). At the peak of vegetative biomass (August), we recorded the species composition, frequency, cover, height, and soil nutrients within each plot in 2015 and 2022 to examine short- and long-term effects, respectively. Plant species are identified and classified with reference to “Flora Reipublieae Popularis Sinicae” (http://www.iplant.cn/frps).

### 2.3 Statistical analysis

The importance value is a useful measure for species location in a community and is used to indicate the population dominance. The dominant species in the community with importance values more than 0.5% were selected for niche analysis (Camargo et al., 2021). The importance value is calculated as follows (**Tables 1**, **2**):

**Table 1:** The dominant species Levins niche width for PFGs treatments in 2015 and 2022.

PFGs	Dominant species	Treatments									
		2015					2022				
		CK	RG	RC	RL	RF	CK	RG	RC	RL	RF
Gramineae	*Stipa aliena*	4.99	/	2.99	4.98	3.99	/	/	/	/	/
	*Deschampsia cespitosa*	4.98	/	3.02	4.99	3.98	/	/	/	/	/
	*Elymus nutans*	4.99	/	2.99	4.93	3.98	/	/	/	/	/
	*Poa araratica*	5.02	/	2.99	4.89	3.99	1.00	/	1.56	1.00	1.93
	*Helictotrichon tibeticum*	5.00	/	2.98	5.00	4.00	1.99	/	2.90	1.99	2.25
Cyperaceae	*Trichophorum distigmaticum*	3.99	2.99	/	4.95	3.94	/	/	/	/	/
	*Carex parva*	4.99	4.91	/	4.98	3.96	4.02	3.55	/	2.90	2.82
	*Carex alatauensis*	3.98	4.99	/	4.90	3.98	2.85	1.76	/	2.65	3.61
Legumes	*Oxytropis arctica*	2.99	4.62	2.99	/	3.97	2.43	2.54	4.29	/	4.68
	*Medicago ruthenica*	2.97	3.98	2.00	/	3.94	4.79	3.81	3.73	/	3.78
	*Gueldenstaedtia verna*	4.89	4.77	3.00	/	2.00	/	/	/	/	/
	*Tibetia himalaica*	/	/	/	/	/	2.93	1.00	1.38	/	1.91
Forbs	*Saussurea pulchra*	3.00	3.45	2.99	3.99	/	3.57	2.88	4.41	3.24	/
	*Gentiana aristata*	4.97	2.75	3.00	3.98	/	4.69	1.99	1.41	1.84	/
	*Gentiana straminea*	4.97	4.89	1.99	3.96	/	3.94	2.35	3.69	3.37	/
	*Thalictrum alpinum*	3.96	3.82	3.00	4.99	/	4.71	3.95	3.96	3.56	/
	*Ranunculus pulchellus*	2.99	4.96	1.99	3.93	/	2.71	2.83	4.55	4.57	/
	*Potentilla nivea*	4.00	4.07	1.98	3.90	/	3.33	3.42	4.84	3.07	/
	*Argentina anserina*	1.98	3.39	1.00	4.00	/	/	/	/	/	/
	*Lancea tibetica*	4.00	3.99	1.00	3.99	/	4.37	3.13	3.85	2.93	/
	*Sibbaldianthe bifurca*	1.99	2.73	1.99	1.00	/	4.72	2.97	2.80	2.00	/
	*Taraxacum mongolicum*	/	/	/	/	/	2.87	2.46	1.35	4.22	/
	*Anemone obtusiloba*	/	/	/	/	/	3.46	2.38	1.00	1.00	/
	*Potentilla multifida*	/	/	/	/	/	3.43	3.35	3.53	3.61	/

**Table 2:** Interspecific association of dominant species for PFGs treatments in 2015 and 2022.

Treatments	*VR*		*W*		[χ^2^_0.95_, χ^2^_0.05_]		*P*-values	
	2015	2022	2015	2022	2015	2022	2015	2022
CK	0.717	0.419	3.584	2.096	[9.39, 28.87]	[8.67, 27.59]	*P* < 0.05	*P* < 0.05
RG	1.723	0.432	8.618	2.162	[5.89, 22.36]	[7.26, 25.00]	*P* > 0.05	*P* < 0.05
RC	0.923	0.462	4.617	2.311	[7.96, 26.30]	[7.26, 25.00]	*P* < 0.05	*P* < 0.05
RL	0.389	0.503	1.948	2.514	[7.96, 26.30]	[6.57, 23.68]	*P* < 0.05	*P* < 0.05
RF	0.014	1.437	0.071	7.186	[3.33, 16.92]	[1.64, 12.59]	*P* < 0.05	*P* > 0.05


$$\begin{array}{c} \text{Importance value }\\ =\;\left(\text{relative density }+\text{ relativecover }+\text{ relative frequency}\right)/3 \end{array}$$

The Levins index (*B_L_*) was used to determine niche width (Lu et al., 2020), and the Pianka index (*O_ik_*) was used to calculate niche overlap (Schoener, 1974). These indices were calculated as follows:


$$B_{L}=1/\sum_{j=1}^{r}P_{ij}^{2}$$

$$O_{ik}=\frac{\sum_{j=1}^{r}p_{ij}p_{kj}}{\sqrt{\sum_{j=1}^{r}p_{ij}^{\;2}\sum_{j=1}^{r}p_{kj}^{\;2}\;}}$$

where *P_i_*_j_ and *P_kj_* are the importance values of species *i* and species *k* within the *j^th^* plot, respectively; and *O_ik_* is the niche overlap index between species *i* and *k*, ranging from 0 to 1.

The variance ratio (*VR*) test was used to determine the overall interspecific association at the community level. *VR* indicates whether there is a significant relationship among multiple species in the selected area, and its significance was tested using the *W* statistic (Schluter, 1984). *VR* was calculated as follows:


$$VR=\frac{\frac{1}{N}\sum_{i=1}^{N}{\left(T_{i}-t\right)}^{2}}{\sum_{j=1}^{S}\left(1-P_{i}\right)}$$

$$P_{j}=\frac{n_{j}}{N}$$

where *N* is the total number of plots in the community, *T_i_* is the total number of target species in plot *i*, *t* is the mean number of species observed in all plots, *S* represents the total number of species in the community, *P_j_* represents the frequency of species *j*, and *n_j_* represents the total number of plots occupied by species *j* (Schluter, 1984).

Under the null hypothesis of independence, the expected value of *VR* is 1; that is, when *VR* = 1, it means that there is no connection between the species. If *VR* > 1, then it means that there is a positive connection between the species. If *VR* < 1, then it means that there is a negative connection between the species. The statistic *W* was used to verify the significant degree of *VR* deviation from 1, W = VR × N. If W > χ^2^_0.05_ (N) or W < χ^2^_0.95_ (N), then it means that the overall connection between the species is significant. On the contrary, the overall connection between the species is not significant (Wang et al., 2018).

Interspecific association has been widely used in interspecific ecological studies to determine the significance of interspecific association by using the χ^2^ test, to determine interspecific associations with the Association coefficient (*AC*), and to analyze the interspecific associations strength based on the Ochiai index (*OI*), and this methodology has also been applied to the study of interspecific relationships in grassland plants (Zhang et al., 2018; Liu et al., 2019). Pairwise interspecific association is one of the important quantitative and structural characteristics of plant communities, which is the basis for the formation and evolution of plant community structure (Zhong et al., 2010). *OI* analysis of the strength of connectivity between species pairs (Zhang et al., 2018). The χ^2^ value, *AC*, and *OI* were calculated as follows:


$$x^{2}=\frac{N{\left(\left|ab-bc\right|-0.5N\right)}^{2}}{\left(a+b\right)\left(c+d\right)\left(a+c\right)\left(b+d\right)}$$

$$\text{AC}=\frac{\text{ad}-\text{bc}}{\left(\text{a}+\text{b}\right)\left(\text{b}+\text{d}\right)}\;(\text{ad}>\text{bc})$$

$$AC=\frac{\text{ad}-\text{bc}}{\left(\text{a}+\text{b}\right)\left(\text{a}+\text{c}\right)}\;(\text{bc}>\text{ad},\text{ d}\geq \text{a})$$

$$AC=\frac{\text{ad}-\text{bc}}{\left(\text{a}+\text{b}\right)\left(\text{d}+\text{c}\right)}\;(\text{bc}>\text{ad},\text{ d}>\text{a})$$

$$OI=\frac{a}{\sqrt{\left(a+b\right)\left(a+c\right)}}$$

where *N* represents the total number of quadrats, α represents the number of quadrats with two species, b and c represent the number of quadrats with only one species, whereas d represents the number of quadrats with neither of the two species. The interspecific association is not significant when χ^2^ < 3.814 (*P* > 0.05), at which point it is considered largely independent; when χ^2^ > 3.814, at which point it is considered significant (*P* < 0.05); when ad > bc, the interspecific association is considered positive, and vice versa.

All analyses were conducted in the R v4.2.1 statistical platform. The niche width, niche overlap, community-level interspecific association, *AC*, *OI*, and statistical analyses were performed using the “*spaa*” package, and semi-matrix plots were performed using the “*ggplot*” package.

## 3 Results

### 3.1 Response of dominant species niche width to PFG removal

**Figure 2:**
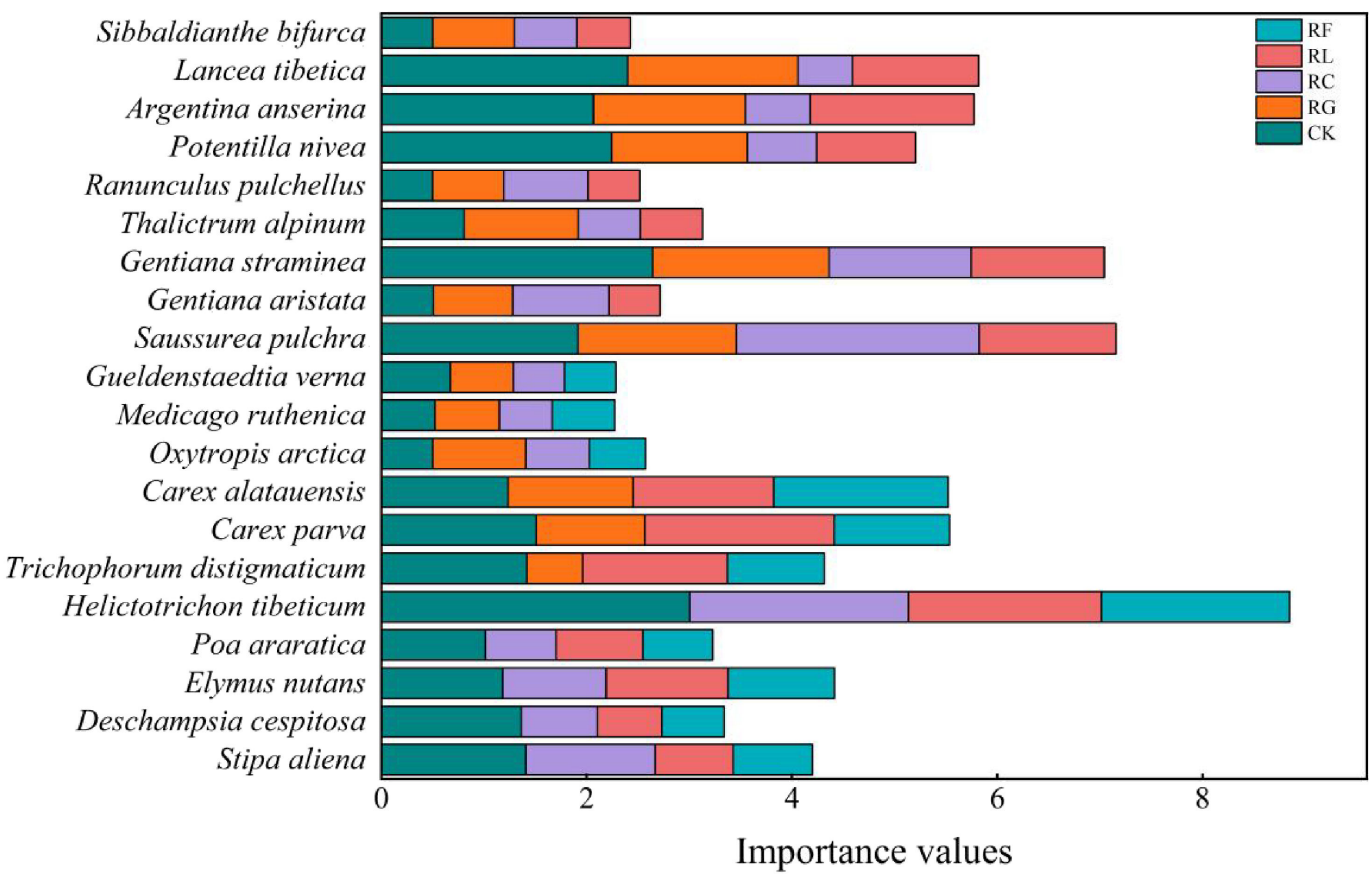
Importance values for dominant species for PFGs treatments in 2015.

**Figure 3:**
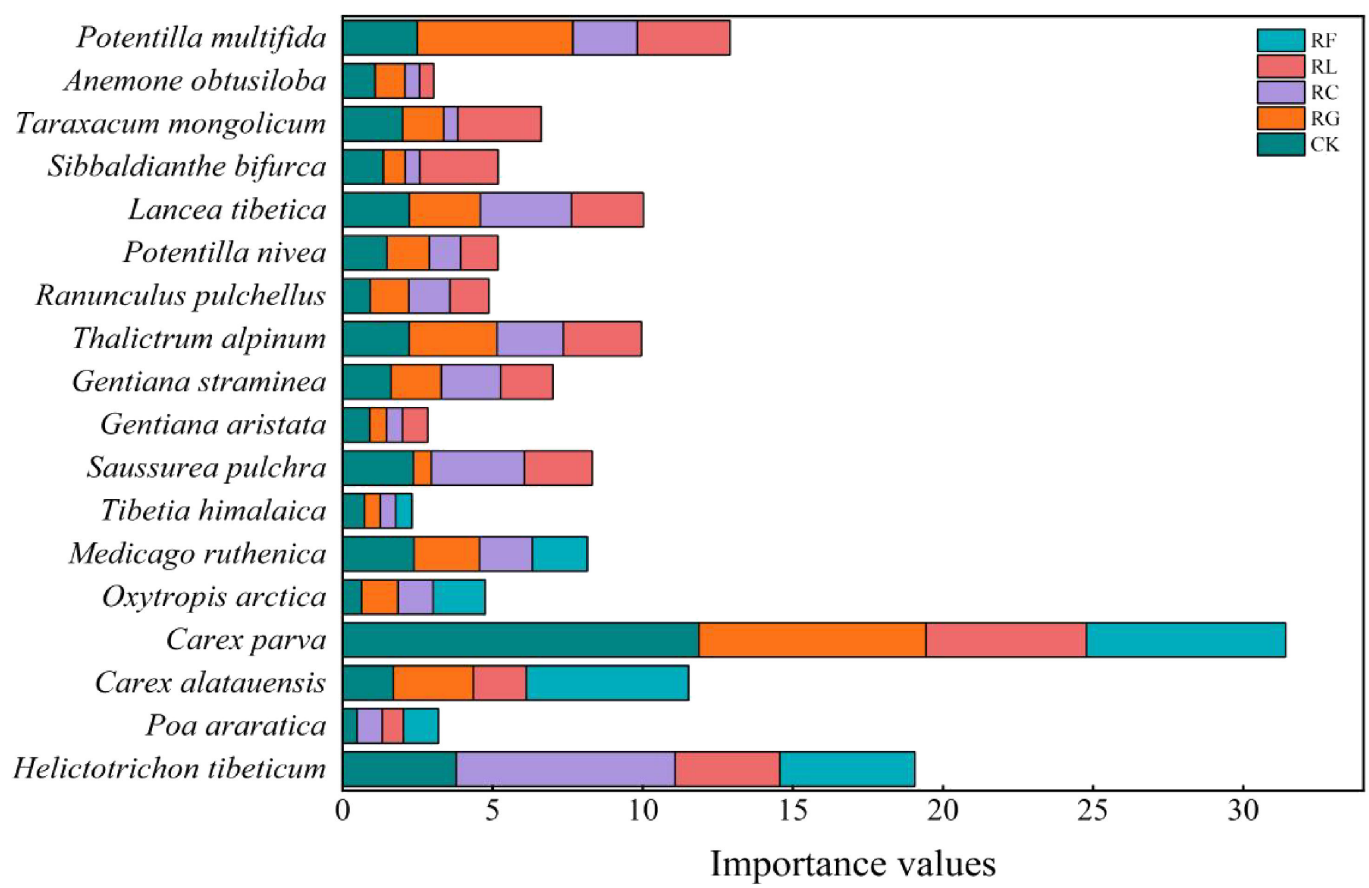
Importance values for dominant species for PFGs treatments in 2022.

Differences in the dominant species niche widths within the same experimental treatments at different time scales (**Figures 2**, **3**; **Table 1**). In the short term (2015), there were 20 dominant species in the study area, including 5 Gramineae, 3 Cyperaceae, 3 legumes, and 9 other forbs. In the long term (2022), there were 18 dominant species in the study area, including 2 Gramineae, 2 Cyperaceae, 3 legumes, and 11 other forbs. The species with the niche width range in the short-term removal period was *Poa araratica* (5.02) in the control, *Carex alatauensis* (4.99) in the RG treatment, and *Deschampsia cespitosa* (3.02) in the RC treatment. The species with the greatest niche width in the RL treatment was *Helictotrichon tibeticum* (5.00), and, in the RF treatment, it was *Helictotrichon tibeticum* (4.00). In the long-term removal period, the species with the niche width range was *Sibbaldianthe bifurca* (4.72) in the control, *Thalictrum alpinum* (3.95) in the RG treatment, *Potentilla nivea* (4.84) in the RC treatment, *Ranunculus pulchellus* (4.57) in the RL treatment, and *Oxytropis arctica* (4.68) in the RF treatment. The maximum niche wide of the species in each treatment was lower in the short term compared to that in the control, whereas there was no regular change in the long term.

### 3.2 Response of dominant species niche overlap to PFG removal

**Figure 4:**
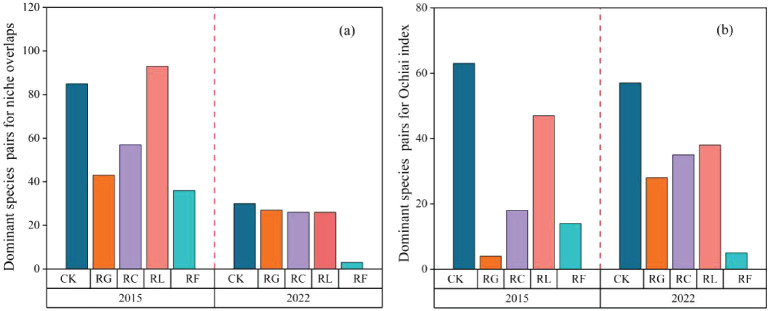
The dominant species pairs for niche overlaps **(A)** and Ochiai index **(B)** for PFGs treatments in 2015 and 2022.

Differences in the dominant species niche overlaps after PFG removal (**Figure 4A**; **Supplementary Figure S1**). The species pairs with maximum niche overlap during the short-term removal were 85 for the control, 43 species pairs for the RG treatment, 57 species pairs for the RC treatment, 93 species pairs for the RL treatment, and 36 species pairs for the RF treatment. For the long-term removal, the species pairs with maximum niche overlap were 30 for the control, 27 species pairs for the RG treatment, 26 species pairs for the RC treatment, 26 species pairs for the RL treatment, and 3 species pairs for the RF treatment. Overall, the number of species pairs with maximum niche overlap decreased as removal time proceeded.

### 3.3 Response of dominant species interspecific association to PFG removal

We evaluated overall interspecific associations between species within the community (**Table 2**). Positive correlations (*VR* > 1) were observed among 14 species pairs in the RG treatment during the short-term time. Similarly, positive correlations (*VR* > 1) were present among seven species pairs in the RF treatment during the long-term time. Conversely, negative correlations were identified among all remaining species pairs in the other treatments (*VR* < 1). The *W* Statistics showed that the overall associations among dominant species in the treatment with negative correlations among species pairs were all significant.

The number of species pairs showing a positive correlation in the short-term removal time varied among treatments (**Table 3**; **Supplementary Figure S2**). There were 89 species pairs (46.84%) in the control, 64 species pairs (60.95%) in the RG treatment, 101 species pairs (74.26%) in the RC treatment, 72 species pairs (52.94%) in the RL treatment, and 25 species pairs (45.45%) in the RF treatment. In the long-term removal time, the number of positively correlated species pairs were 72 (47.06%) in the control, 55 (45.83%) in the RG treatment, 61 (50.83%) in the RC treatment, 57 (54.28%) in the RL treatment, and 12 (57.14%) in the RF treatment. All other species pairs exhibited a negative correlation (**Table 3**; **Supplementary Figure S2**). The number of species pairs that were positively correlated across treatments decreased with time of removal compared to the control.

**Table 3:** Correlated species pairs of dominant species for PFGs treatments in 2015 and 2022.

Treatments	2015		2022	
	Positive	Negative	Positive	Negative
CK	89	101	72	81
RG	64	41	55	65
RC	101	35	61	59
RL	72	64	57	48
RF	25	30	12	9

### 3.4 Response of dominant species association coefficient to PFG removal

In the short-term removal time, the number of species pairs within the maximum AC range among the dominant species in the treatments followed the order of CK (21) > RL (5) > RF (4) = RC (4) > RG (2) (**Supplementary Figure S3**). In the long-term removal time, the number of species pairs within the maximum AC range of the dominant species in the treatments followed the order of RL (13) = RG (13) > RC (6) > CK (4) > RF (1) (**Supplementary Figure S3**). Under the highest interspecific association coefficients, the CK and RL treatment had the highest number of species pairs within the community, and the niches between species were more complex.

### 3.5 Response of dominant species Ochiai index to PFG removal

The number of species pairs present in the conditions with the highest values of interspecific linkage strength varied among treatments (**Figure 4B**; **Supplementary Figure S4**). There were 63 species pairs in the control, 4 species pairs in the RG treatment, 18 species pairs in the RC treatment, 47 species pairs in the RL treatment, and 14 species pairs in the RF treatment during the short-term removal time. In the long-term removal time, there are 57 species pairs in the control, 28 species pairs in the RG treatment, 35 species pairs in the RC treatment, 38 species pairs in the RL treatment, and 5 species pairs in the RF treatment.

## 4 Discussion

### 4.1 Effects of PFG removal on niche width and niche overlap

Niche width reflects the position and role of a population within a community (Carscadden et al., 2020). In this study, the number of dominant species differed across the experimental treatments, with a decrease in the number of Gramineae and Cyperaceae and an increase in the number of forbs other than legumes. Thus, long-term species diversity loss led to gradual reductions in the niche widths of Gramineae and Cyperaceae, slowly disadvantaging them in terms of resource competition. By contrast, the niche width of other forbs gradually increased, enhancing their competitiveness for resources. Our results also provided strong evidence of alpine meadow degradation. The present findings are consistent with a previous report that the combined dominance ratios of Gramineae, Cyperaceae, and forbs other than legumes are negatively correlated (Jiang et al., 2021). In this study, only when other forbs were removed did the remaining Gramineae and Cyperaceae attain larger niche widths. The niche widths of the remaining functional group treatments gradually shifted toward dominance by other forbs, indicating that these forbs were the most powerful competitors for environmental resources in the alpine meadow plant communities. Kong et al. (2011) indicated that PFG removal resulted in less aboveground biomass to provide the photosynthetic products needed to maintain root biomass, thereby affecting root nutrients; thus, the aboveground traits of forbs generally favor the uptake of more environmental resources, which in turn, can affect the survival of other PFGs to a greater extent. In this manner, the presence of forbs may be among the most important factors affecting the survival of other species. The niche width of the remaining PFGs after species loss varied by time scale; generally, longer intervals resulted in more realistic niche widths, as reflected by compensatory effects in the remaining species. Short-term species culling might have caused our results to be influenced by the compensatory effects of the remaining PFGs; because most of the biomass in alpine meadow is allocated to belowground plant parts, the species remaining after culling may not show significant compensation in the short term (Díaz et al., 2003; Bai et al., 2004), such that long-term monitoring would lead to more accurate results.

Niche overlap is a measure of the degree of species similarity in terms of environmental adaptation or resource use (Pastore et al., 2021). In this study, the niche overlap of the remaining dominant species in the community shifted from high to low, and the number of species pairs with the highest niche overlap shifted from high to low as the monitoring period was extended. Thus, our results indicated that species loss caused the remaining species to be less similar in terms of resource use. Previous studies have shown that species coexistence presupposes niche differentiation and that species with identical niches cannot coexist in the long term (Song, 2017); however, niche overlap does not necessarily lead to competition between species and may be necessary for exploitative competition, as competition is also related to resource amounts and population sizes (Letten et al., 2017). The high degree of niche overlap detected in this study cannot directly confirm intense competition between species but rather indicates high potential for competition.

### 4.2 Effects of PFG removal on interspecific association

The overall connectivity between community species is closely related to stability (Wittebolle et al., 2009). Based on previous studies, during positive community succession, species maximize resource use through competition and cooperation, species composition is gradually fixed, community structure becomes more stable and eventually reaches an apex, and coexisting species tend to be positively associated; however, the association between individual species pairs decreases over the course of succession (Guo et al., 2008; Haugaasen and Peres, 2009). In this study, overall species connectivity remained negative during the 10-year monitoring trial; however, overall species connectivity was positive in the treatment in which forbs other than legumes were completely removed, probably because Gramineae and Cyperaceae are dominant species in alpine meadows and thus determine the development direction of the community to some extent. As such, the community examined in this study may have reached a relatively stable late successional stage.

Interspecific associativity reflects interrelationships between the species comprising a community over a period of time (Ru et al., 2022). It is often assumed that positively associated species pairs have mutually beneficial and complementary relationships, whereas negatively associated species pairs are more likely to experience competition and exclusion (Levine et al., 2017). As time passes, most of the treatment trials showed negative associations, suggesting that species interactions tend to be mainly competitive and exclusionary; however, the treatment in which other forbs were completely removed showed the opposite trend, toward mutualism and complementarity, indicating that species interactions under this treatment were dominant in terms of resource use (Åkesson et al., 2021). Interspecific correlations are based on multidegree data between species pairs and can provide a basis for analyzing species extinction patterns within a community (Norberg et al., 2012). These species interrelationships are consistent with interspecific associations but differ somewhat in terms of sensitivity. Generally, when two species appear or disappear simultaneously in a sample, the association between these species is positive; however, the association could be negative if the number of individuals of one species increases while that of the other species decreases (Liu et al., 2019). In our study, this difference was reflected by the higher proportion of negatively correlated species pairs detected by the Pearson correlation test than by the χ^2^ test and AC values in this study.

### 4.3 Effects of PFG removal on niche construction

It is increasingly recognized that all organisms modify their environments (i.e., niche construction or ecosystem engineering) in response to various environmental disturbances (Odling-Smee et al., 2013). Such changes include effects on the distribution and abundance of organisms, dominant species, control of energy, material flows, ecosystem resilience, and specific nutrient relationships (Chapin et al., 1997). There is evolutionary evidence that, even when key resources are independently renewed or depleted, the effects of niche construction can override selection from external sources, thereby creating new evolutionary trajectories and equilibria, generating and eliminating polymorphisms, and producing time-lagged selection as well as other unusual dynamic responses (Laland et al., 1999). The present study revealed that long-term species loss resulted in larger niche widths for forbs other than legumes and smaller niche widths for dominant species in the community. Furthermore, niche overlapping pairs among dominant species in the community tended to decrease with removal treatment time, and the number of related species pairs decreased, indicating that competition intensity between species within the community tended to weaken. Interestingly, competitive intensity between species became stronger or weaker more rapidly when forbs other than legumes were removed from the community. The removal of different PFGs in this study was considered to reflect the process of reconstructing the niche of each species within the community under temporal turnover, including resource acquisition, resource use within changing communities, and interactions between species. Further investigation is required to explore how niche reconstruction among species in communities occurs over time, the key channels through which such reconstruction occurs, and changing patterns in the construction of linkages among species.

The niche overlap pairs of dominant species in each treatment were fewer compared to the control, and the niche overlap value in the control changed with the delay. The community’s species relationships exhibited a decline in positively correlated species pairs over time, with both the number of these pairs and the strength of associations in each treatment being lower than those in the control. Species interactions can both mitigate and exacerbate the effects of climate change on species and interact with other eco-evolutionary processes (Post, 2013). For example, species interactions can affect the evolutionary response to altered environmental conditions; dispersal can release species from negative interactions or increase them through migration or through invasions (Mazancourt et al., 2008; Walther et al., 2009; Van Eldijk et al., 2020). However, this phenomenon is not certainly caused by changes in the environment but is closely related to the number of potential interspecific competitors in the community or the so-called “diffuse competition,” and it has been argued that the maximum tolerable overlap should be inversely proportional to the intensity of the competition, which is really the main content of the niche overlap hypothesis (Pianka, 1974). More importantly, most of the studies have been conducted under fenced conditions, and the results of the experiments have limitations. However, an increasing number of studies pointed out that the species niches are affected by space and time and that quantifying species niches is needed to explore the relationship between species distributions and environment, as well as to predict the potential risk of species invasions and future species extinction rates (Jackson et al., 2009; Liu et al., 2020). In the future, we need to further explore the effects of plant diversity loss on species niches and ecosystem functioning at broader spatial scales and longer time scales.

## 5 Conclusions

Our results indicated that removal of different PFGs affected plant community composition and niche dynamics. After 3 and 10 years of experiments, the species composition of the community gradually shifted from being dominated by Gramineae and Cyperaceae to other forbs. Compared to the short term, long-term PFG removal led to narrower niche widths for Gramineae and Cyperaceae, reduced community niche overlap, and a trend toward fewer positively related species. In the case of environmental resource use, the decreased niche overlap indicates interspecific competition within the community, albeit decreasing. In summary, reduced species diversity resulted in significant niche differences among the remaining species in the community, shifting resource allocation from advantaged to disadvantaged species. Therefore, we emphasize the importance of ongoing long-term PFG removal experiments and studies that investigate a wider range of domains across broader spatial and temporal scales to achieve a more accurate and refined response mechanism.

## Data Availability

The raw data supporting the conclusions of this article will be made available by the authors, without undue reservation.
