# Supplementary material for: Effects of short- and long-term plant functional group removal on alpine meadow community niche
Source: Front Plant Sci. 2024 Nov 14;15:1474272. doi: 10.3389/fpls.2024.1474272 (PMC11602315; doi:10.3389/fpls.2024.1474272)
Supplement: Supplementary file 1 [file DataSheet1.pdf]

Figure S1. Niche overlap of dominant species from different PFG treatments in 2015 and 2022. A, *Stipa aliena Keng*; B, *Deschampsia cespitosa*; C, *Elymus nutans*; D, *Poa araratica*; E, *Helictotrichon tibeticum*; F, *Trichophorum distigmaticum*; G, *Carex parva*; H, *Carex alatauensis*; I, *Oxytropis arctica*; J, *Medicago ruthenica*; K, *Gueldenstaedtia verna*; L, *Saussurea pulchra*, M, *Gentiana aristata*; N, *Gentiana straminea*; O, *Thalictrum alpinum*; P, *Ranunculus pulchellus*; Q, *Potentilla nivea*; R, *Argentina anserina*; S, *Lancea tibetica*; T, *Sibbaldianthe bifurca*; 1, *Helictotrichon tibeticum*; 2, *Poa araratica*; 3, *Carex alatauensis*; 4, *Carex parva*; 5, *Oxytropis arctica*; 6, *Medicago ruthenica*; 7, *Tibetia himalaica*; 8, *Saussurea pulchra*; 9, *Gentiana aristata*; 10, *Gentiana straminea*; 11, *Thalictrum alpinum*; 12, *Ranunculus pulchellus*; 13, *Potentilla nivea*; 14, *Lancea tibetica*; 15, *Sibbaldianthe bifurca*; 16, *Taraxacum mongolicum*; 17, *Anemone obtusiloba*; 18, *Potentilla multifida*.

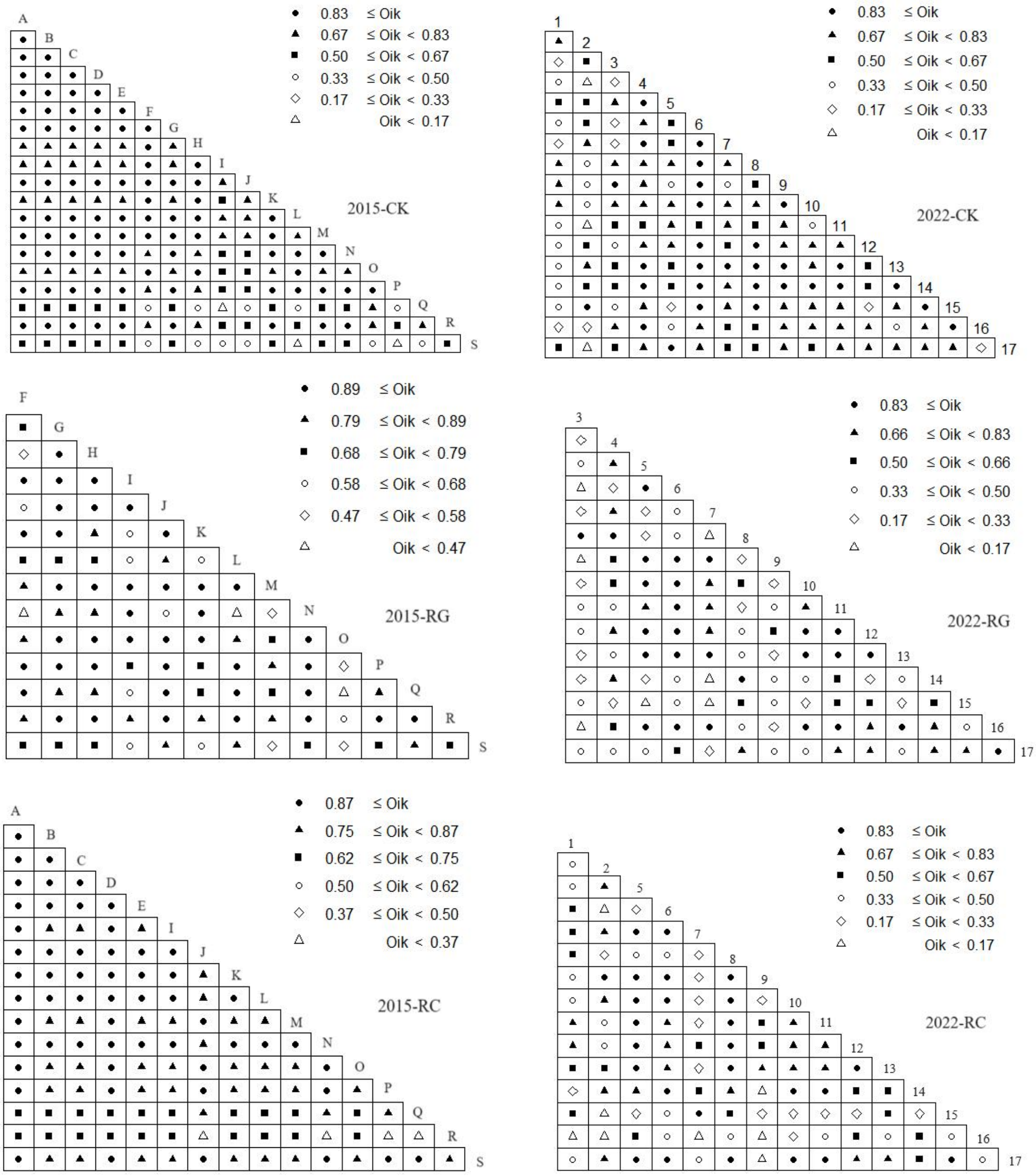

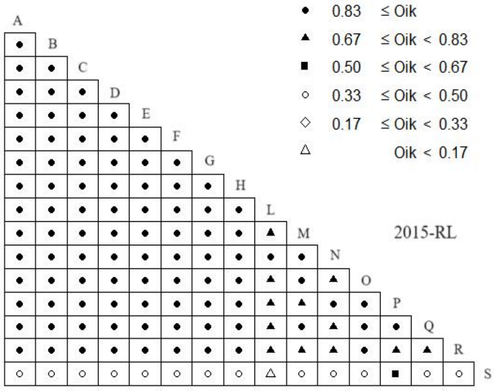
0.83 ≤ Oik
0.67 ≤ Oik < 0.83
0.50 ≤ Oik < 0.67
0.33 ≤ Oik < 0.50
0.17 ≤ Oik < 0.33
Oik < 0.17
2015-RL
A B C D E F G H L M N O P Q R S

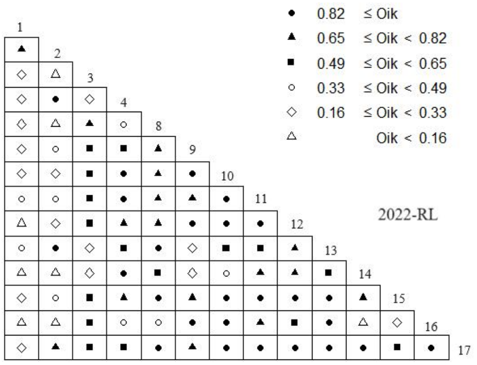
0.82 ≤ Oik
0.65 ≤ Oik < 0.82
0.49 ≤ Oik < 0.65
0.33 ≤ Oik < 0.49
0.16 ≤ Oik < 0.33
Oik < 0.16
2022-RL
1 2 3 4 8 9 10 11 12 13 14 15 16 17

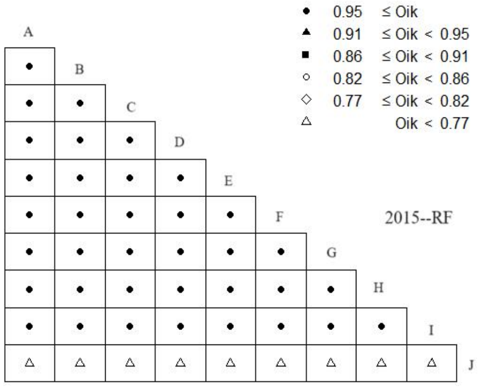
0.95 ≤ Oik
0.91 ≤ Oik < 0.95
0.86 ≤ Oik < 0.91
0.82 ≤ Oik < 0.86
0.77 ≤ Oik < 0.82
Oik < 0.77
2015--RF
A B C D E F G H I J

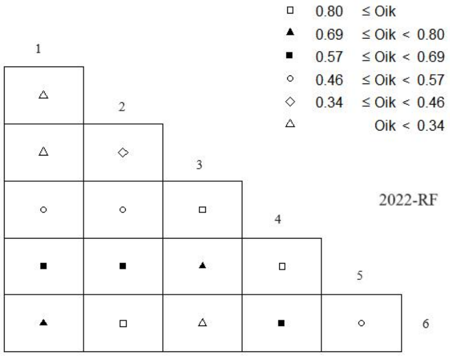
0.80 ≤ Oik
0.69 ≤ Oik < 0.80
0.57 ≤ Oik < 0.69
0.46 ≤ Oik < 0.57
0.34 ≤ Oik < 0.46
Oik < 0.34
2022-RF
1 2 3 4 5 6

Figure S2. *P*-value matrix for chi-square test for overall connectivity of dominant species from different PFG treatments in 2015 and 2022. Blue and a slash from bottom left to top right indicate that the two variables are positively correlated, while red and a slash from top left to bottom right indicate that the variables are negatively correlated. The darker the colour, the stronger the correlation between the variables. A, *Stipa aliena Keng*; B, *Deschampsia cespitosa*; C, *Elymus nutans*; D, *Poa araratica*; E, *Helictotrichon tibeticum*; F, *Trichophorum distigmaticum*; G, *Carex parva*; H, *Carex alatauensis*; I, *Oxytropis arctica*; J, *Medicago ruthenica*; K, *Gueldenstaedtia verna*; L, *Saussurea pulchra*, M, *Gentiana aristata*; N, *Gentiana straminea*; O, *Thalictrum alpinum*; P, *Ranunculus pulchellus*; Q, *Potentilla nivea*; R, *Argentina anserina*; S, *Lancea tibetica*; T, *Sibbaldianthe bifurca*; 1, *Helictotrichon tibeticum*; 2, *Poa araratica*; 3, *Carex alatauensis*; 4, *Carex parva*; 5, *Oxytropis arctica*; 6, *Medicago ruthenica*; 7, *Tibetia himalaica*; 8, *Saussurea pulchra*; 9, *Gentiana aristata*; 10, *Gentiana straminea*; 11, *Thalictrum alpinum*; 12, *Ranunculus pulchellus*; 13, *Potentilla nivea*; 14, *Lancea tibetica*; 15, *Sibbaldianthe bifurca*; 16, *Taraxacum mongolicum*; 17, *Anemone obtusiloba*; 18, *Potentilla multifida*.

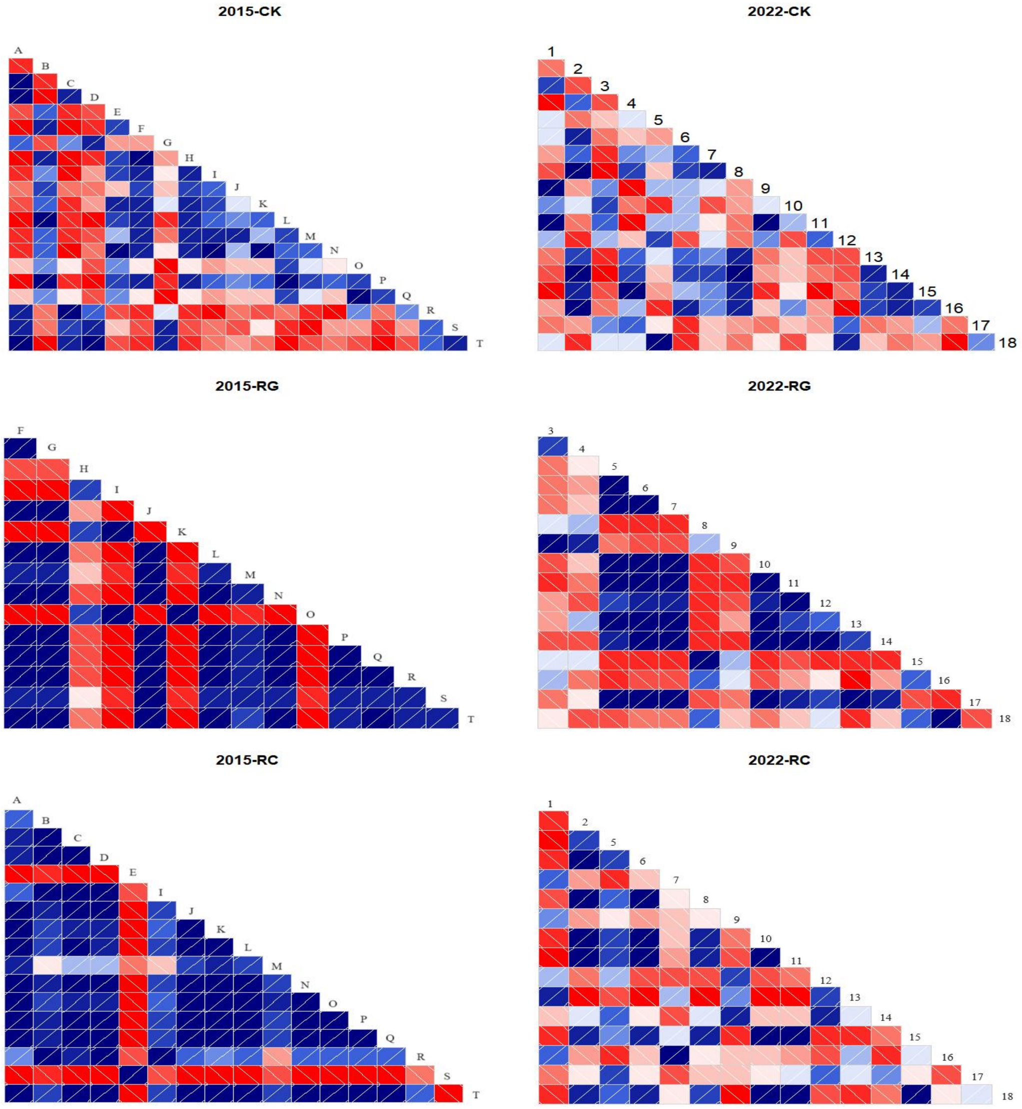

2015-RL

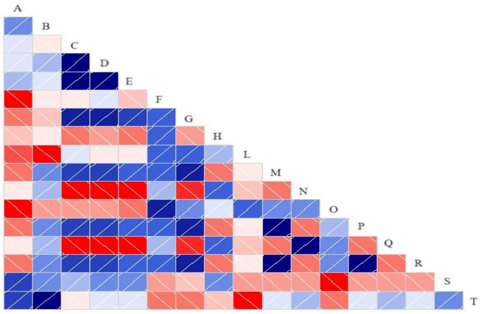

2022-RL

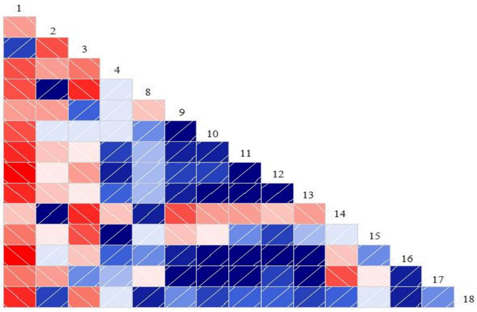

2015-RF

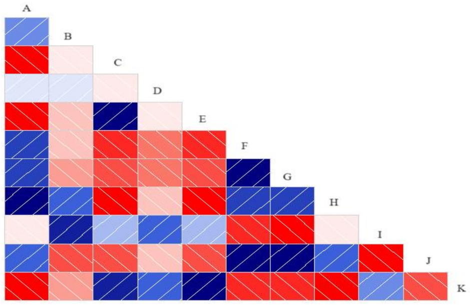

2022-RF

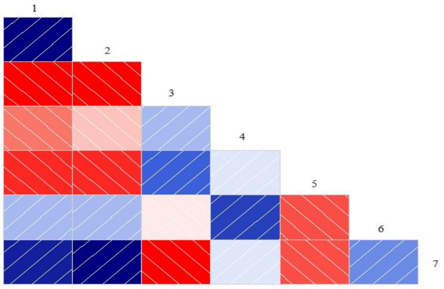

Figure S3. Linkage coefficients of dominant species within the community from different PFG treatments in 2015 and 2022. A, *Stipa aliena Keng*; B, *Deschampsia cespitosa*; C, *Elymus nutans*; D, *Poa araratica*; E, *Helictotrichon tibeticum*; F, *Trichophorum distigmaticum*; G, *Carex parva*; H, *Carex alatauensis*; I, *Oxytropis arctica*; J, *Medicago ruthenica*; K, *Gueldenstaedtia verna*; L, *Saussurea pulchra*, M, *Gentiana aristata*; N, *Gentiana straminea*; O, *Thalictrum alpinum*; P, *Ranunculus pulchellus*; Q, *Potentilla nivea*; R, *Argentina anserina*; S, *Lancea tibetica*; T, *Sibbaldianthe bifurca*; 1, *Helictotrichon tibeticum*; 2, *Poa araratica*; 3, *Carex alatauensis*; 4, *Carex parva*; 5, *Oxytropis arctica*; 6, *Medicago ruthenica*; 7, *Tibetia himalaica*; 8, *Saussurea pulchra*; 9, *Gentiana aristata*; 10, *Gentiana straminea*; 11, *Thalictrum alpinum*; 12, *Ranunculus pulchellus*; 13, *Potentilla nivea*; 14, *Lancea tibetica*; 15, *Sibbaldianthe bifurca*; 16, *Taraxacum mongolicum*; 17, *Anemone obtusiloba*; 18, *Potentilla multifida*.

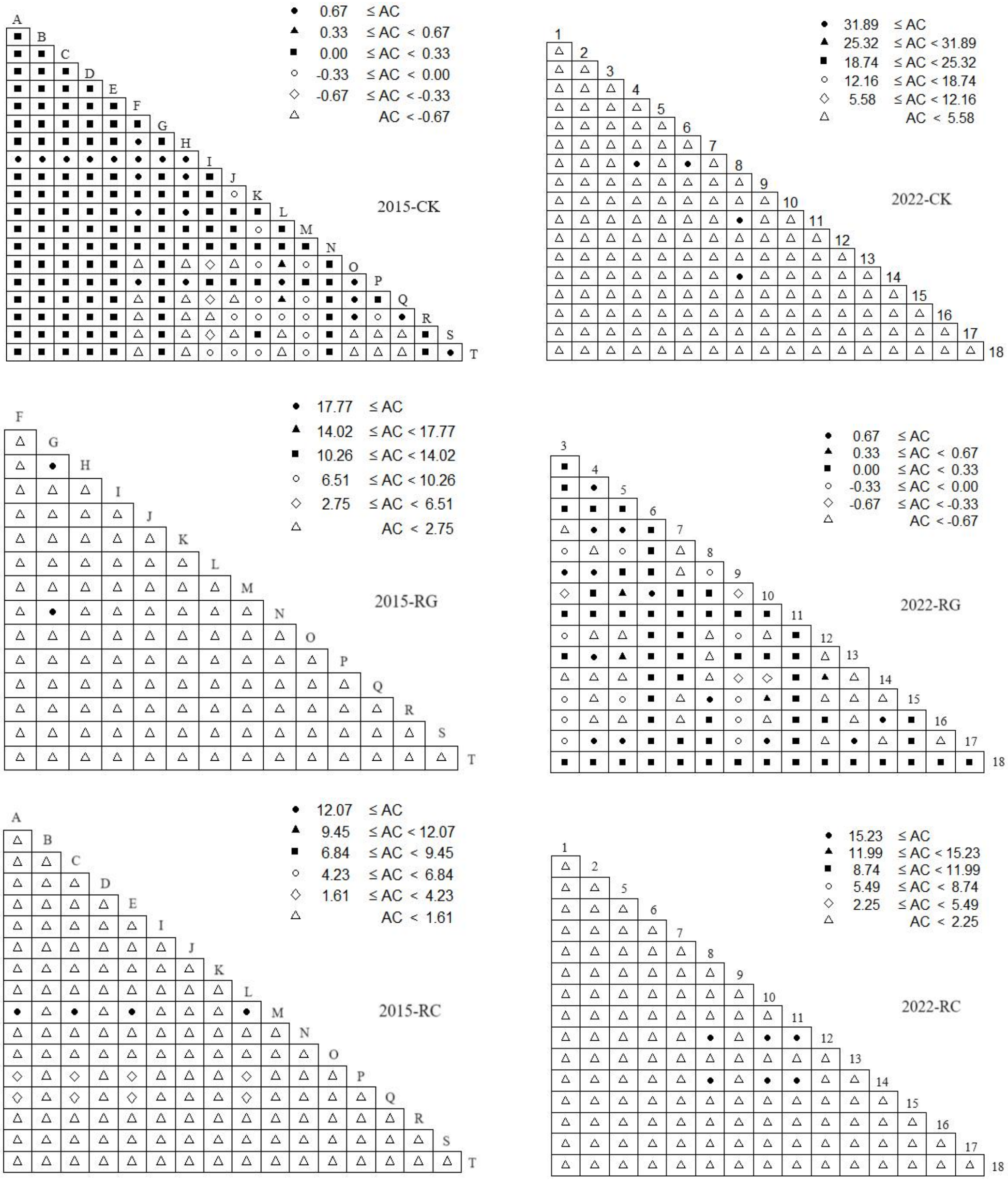

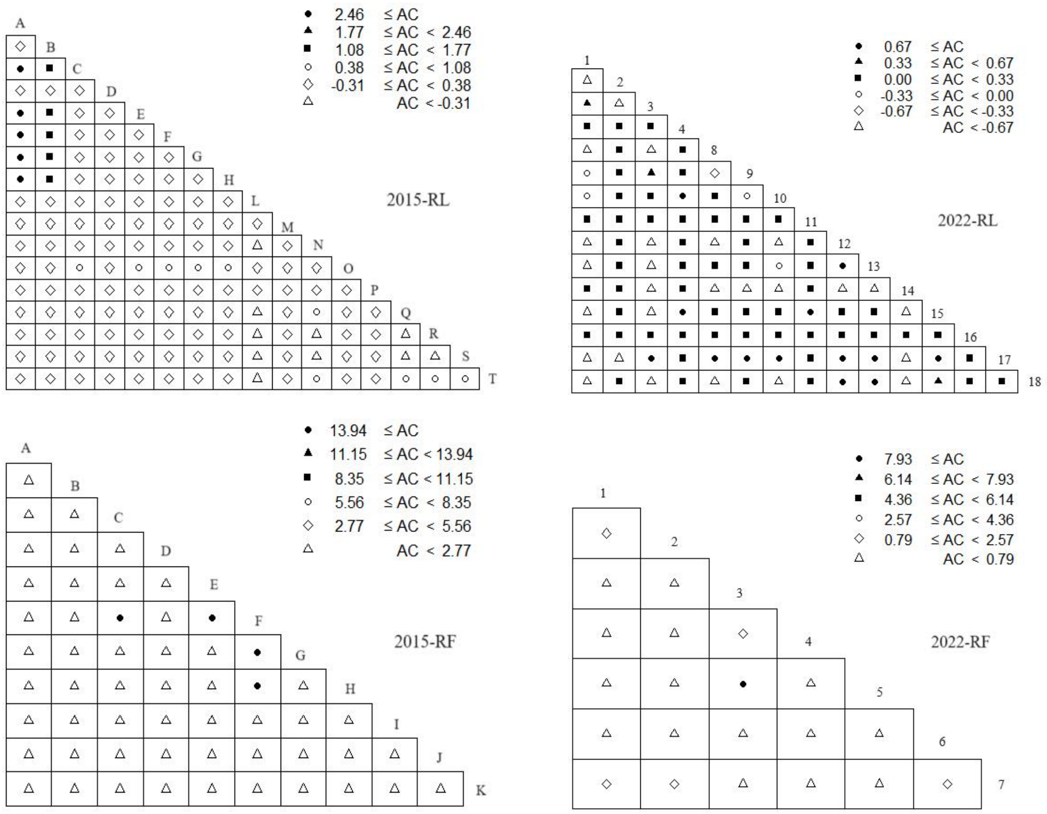
2015-RL
2.46 ≤ AC
1.77 ≤ AC < 2.46
1.08 ≤ AC < 1.77
0.38 ≤ AC < 1.08
-0.31 ≤ AC < 0.38
AC < -0.31
2022-RL
0.67 ≤ AC
0.33 ≤ AC < 0.67
0.00 ≤ AC < 0.33
-0.33 ≤ AC < 0.00
-0.67 ≤ AC < -0.33
AC < -0.67
2015-RF
13.94 ≤ AC
11.15 ≤ AC < 13.94
8.35 ≤ AC < 11.15
5.56 ≤ AC < 8.35
2.77 ≤ AC < 5.56
AC < 2.77
2022-RF
7.93 ≤ AC
6.14 ≤ AC < 7.93
4.36 ≤ AC < 6.14
2.57 ≤ AC < 4.36
0.79 ≤ AC < 2.57
AC < 0.79

Figure S4. Semi-exponential matrix of *OI* for dominant species from different PFG treatments in 2015 and 2022. A, *Stipa aliena Keng*; B, *Deschampsia cespitosa*; C, *Elymus nutans*; D, *Poa araratica*; E, *Helictotrichon tibeticum*; F, *Trichophorum distigmaticum*; G, *Carex parva*; H, *Carex alatauensis*; I, *Oxytropis arctica*; J, *Medicago ruthenica*; K, *Gueldenstaedtia verna*; L, *Saussurea pulchra*, M, *Gentiana aristata*; N, *Gentiana straminea*; O, *Thalictrum alpinum*; P, *Ranunculus pulchellus*; Q, *Potentilla nivea*; R, *Argentina anserina*; S, *Lancea tibetica*; T, *Sibbaldianthe bifurca*; 1, *Helictotrichon tibeticum*; 2, *Poa araratica*; 3, *Carex alatauensis*; 4, *Carex parva*; 5, *Oxytropis arctica*; 6, *Medicago ruthenica*; 7, *Tibetia himalaica*; 8, *Saussurea pulchra*; 9, *Gentiana aristata*; 10, *Gentiana straminea*; 11, *Thalictrum alpinum*; 12, *Ranunculus pulchellus*; 13, *Potentilla nivea*; 14, *Lancea tibetica*; 15, *Sibbaldianthe bifurca*; 16, *Taraxacum mongolicum*; 17, *Anemone obtusiloba*; 18, *Potentilla multifida*.

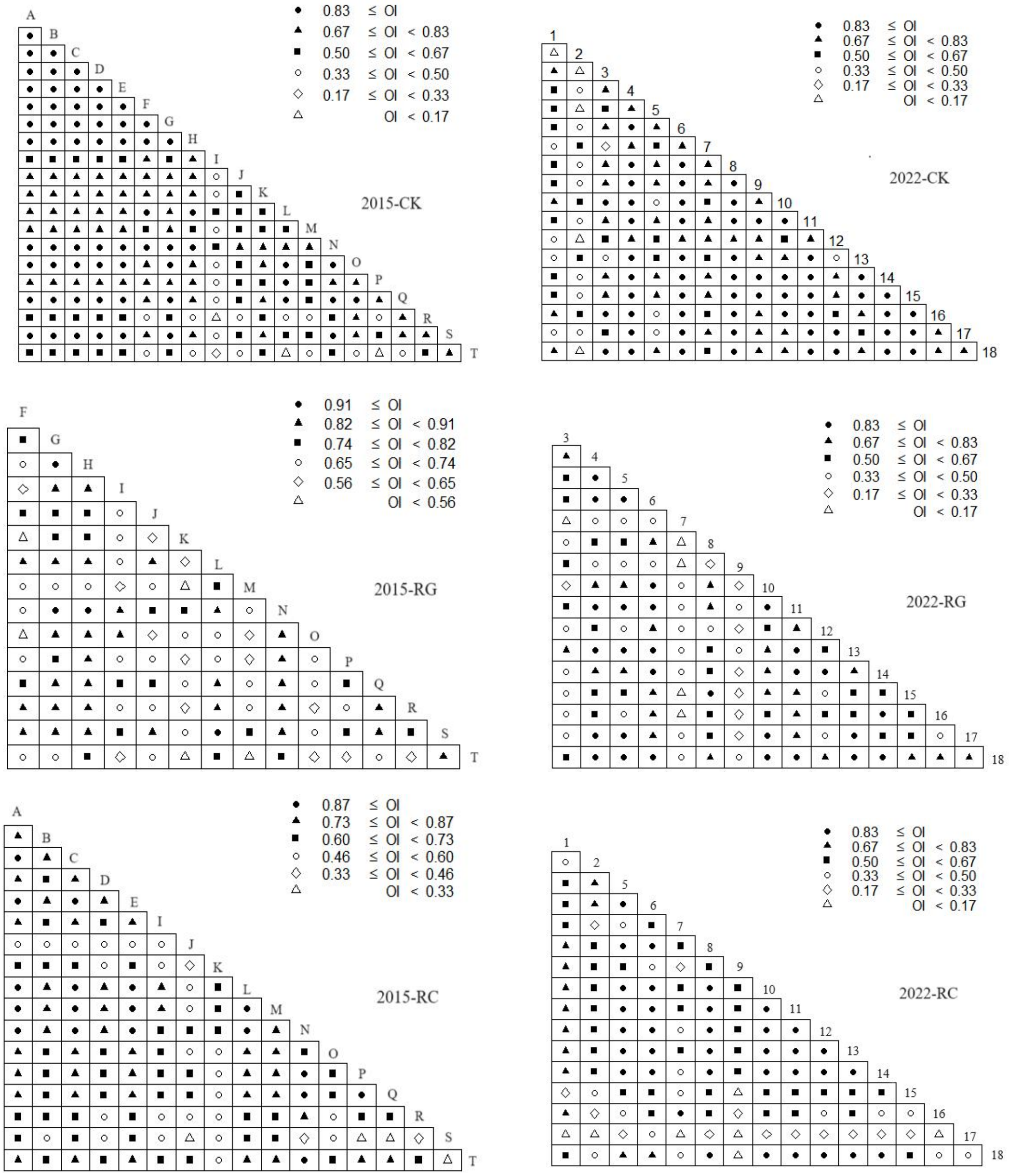

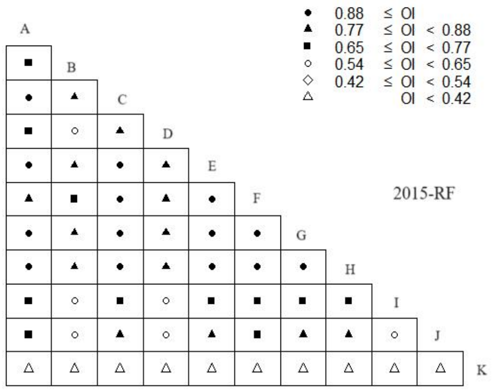
0.88 ≤ OI
0.77 ≤ OI < 0.88
0.65 ≤ OI < 0.77
0.54 ≤ OI < 0.65
0.42 ≤ OI < 0.54
OI < 0.42
2015-RF
A
B
C
D
E
F
G
H
I
J
K

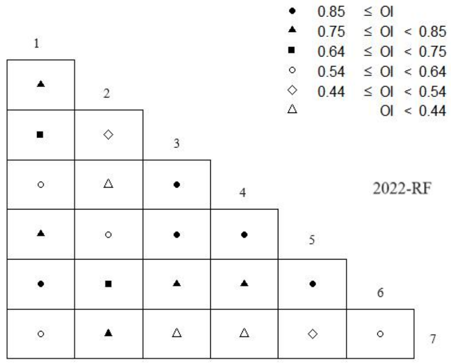
0.85 ≤ OI
0.75 ≤ OI < 0.85
0.64 ≤ OI < 0.75
0.54 ≤ OI < 0.64
0.44 ≤ OI < 0.54
OI < 0.44
2022-RF
1
2
3
4
5
6
7
